# Low Levels of NDRG1 in Nerve Tissue Are Predictive of Severe Paclitaxel-Induced Neuropathy

**DOI:** 10.1371/journal.pone.0164319

**Published:** 2016-10-07

**Authors:** Raghav Sundar, Anand D. Jeyasekharan, Brendan Pang, Richie Chuan Teck Soong, Nesaretnam Barr Kumarakulasinghe, Samuel Guan Wei Ow, Jingshan Ho, Joline Si Jing Lim, David Shao Peng Tan, Einar P. V. Wilder-Smith, Aishwarya Bandla, Stacey Sze Hui Tan, Bernadette Reyna Asuncion, Zul Fazreen, Michal Marek Hoppe, Thomas Choudary Putti, Lay Mui Poh, Boon Cher Goh, Soo-Chin Lee

**Affiliations:** 1 Department of Haematology-Oncology, National University Cancer Institute, Singapore, National University Health System, Singapore, Singapore; 2 Cancer Science Institute of Singapore, National University of Singapore, Singapore, Singapore; 3 Department of Pathology, National University Health System, Singapore, Singapore; 4 Singapore Institute for Neurotechnology (SINAPSE), National University of Singapore, Singapore, Singapore; 5 Department of Medicine, National University Health System, Singapore, Singapore; 6 Department of Medicine, Yong Loo Lin School of Medicine, National University of Singapore, Singapore, Singapore; 7 Department of Biomedical Engineering, National University of Singapore, Singapore, Singapore; 8 Department of Pharmacy, National University Cancer Institute Singapore, National University Health System, Singapore, Singapore; University of Kentucky College of Medicine, UNITED STATES

## Abstract

**Introduction:**

Sensory peripheral neuropathy caused by paclitaxel is a common and dose limiting toxicity, for which there are currently no validated predictive biomarkers. We investigated the relationship between the Charcot-Marie-Tooth protein NDRG1 and paclitaxel-induced neuropathy.

**Methods/Materials:**

Archived mammary tissue specimen blocks of breast cancer patients who received weekly paclitaxel in a single centre were retrieved and NDRG1 immunohistochemistry was performed on normal nerve tissue found within the sample. The mean nerve NDRG1 score was defined by an algorithm based on intensity of staining and percentage of stained nerve bundles. NDRG1 scores were correlated with paclitaxel induced neuropathy

**Results:**

111 patients were studied. 17 of 111 (15%) developed severe paclitaxel-induced neuropathy. The mean nerve NDRG1 expression score was 5.4 in patients with severe neuropathy versus 7.7 in those without severe neuropathy (p = 0.0019). A Receiver operating characteristic (ROC) curve analysis of the mean nerve NDRG1 score revealed an area under the curve of 0.74 (*p* = 0.0013) for the identification of severe neuropathy, with a score of 7 being most discriminative. 13/54 (24%) subjects with an NDRG1 score < = 7 developed severe neuropathy, compared to only 4/57 (7%) in those with a score >7 (*p* = 0.017).

**Conclusion:**

Low NDRG1 expression in nerve tissue present within samples of surgical resection may identify subjects at risk for severe paclitaxel-induced neuropathy. Since nerve biopsies are not routinely feasible for patients undergoing chemotherapy for early breast cancer, this promising biomarker strategy is compatible with current clinical workflow.

## Introduction

Paclitaxel improves survival in early stage breast cancer [1]. Weekly paclitaxel has been shown to be superior in efficacy to the three-weekly regimen [2], albeit at the cost of more severe sensory peripheral neuropathy. Sensory peripheral neuropathy is the most common non-hematological dose limiting toxicity of weekly paclitaxel. Supportive therapy for chemotherapy-induced toxicities has improved significantly over the past few years, particularly in the use of anti-emetics and growth factors for the prevention of nausea and vomiting and hematological toxicities respectively [3, 4]. However, the same cannot be said of chemotherapy induced peripheral neuropathy (CIPN) and it remains an important dose-limiting toxicity of chemotherapy with limited preventive or therapeutic options [5]. Currently there is no recommended treatment for the prevention of CIPN [6]. Several agents have been tested for neuroprotection but none has proven efficacy [7]. The avoidance of potentially disabling chemotherapy-induced peripheral neuropathy (CIPN) is particularly relevant in patients who receive curative cancer treatment as the presence of neuropathy can impair function and impact quality of life. To date, no reliable predictive biomarkers of CIPN exist. The development of a predictive biomarker for drug-induced neuropathy that can readily be measured either in blood or tissue specimens can aid physicians in identifying patients who should avoid neurotoxic chemotherapeutic agents.

We previously performed a single nucleotide polymorphism (SNP) study to analyze the association of several genetic variants with CIPN in an Asian population [8], and found an N-myc downstream regulated gene (NDRG)-1 SNP, rs2233335, to correlate with paclitaxel-induced neuropathy. Mutations in this gene are known to be implicated in autosomal recessively inherited human Charcot-Marie Tooth disease (CMT) type 4D [9], a hereditary motor and sensory neuropathy including peripheral neuropathy. In CMT4D, NDRG1 has been shown to cause degradation of myelin leading to severe neuropathy [10]. We analyzed all NDRG1 SNPs that are in linkage disequilibrium with the rs2233335 SNP, and found all linked SNPs to be localized to the intronic region of the gene, suggesting that splice regulatory elements or other regulatory mechanisms that influence NDRG-1 expression may be implicated. We therefore proceeded to study NDRG1 expression level in human nerve tissues to assess its relationship with paclitaxel induced neuropathy.

Immunohistochemical (IHC) staining of neural proteins such as S100 is commonly performed in clinical practice and is utilized to identify nerve bundles. To the best of our knowledge, testing protein expression directly on normal nerve tissue to predict chemotherapy-induced neuropathy is a novel approach that has never been previously studied. While performing nerve biopsies to predict drug-induced neuropathy is not clinically practical, examining nerve tissue present in resected breast cancer specimens can serve as a surrogate as an abundance of nerve tissue can often be found in these surgical specimens. We present findings of the first study of NDRG1 as a promising protein biomarker in normal nerve tissue found in mastectomy or lumpectomy specimens to predict paclitaxel-induced peripheral neuropathy.

## Methods/Materials

This study was carried out in accordance with the recommendations of the Institutional Review Board (IRB) of the National Health Group, Singapore with ethics approval being provided specifically for this study. All subjects gave written informed consent in accordance with the Declaration of Helsinki for the collection of genetic data and clinical information as part of a larger pharmacogenetics database. As this was a sub-study from the pharmacogenetics database, a separate IRB approval was provided, but waiver of a second informed consent was approved by the IRB. From this database, early stage breast cancer patients who received adjuvant weekly paclitaxel from 2008 to 2015 were included. Only patients who had mastectomies or lumpectomies including the nipple were included in the study. All patients selected for the study were planned for 12 cycles of continuous weekly paclitaxel at 80mg/m^2^ for a total of 960 mg/m^2^ unless they developed toxicities requiring dose reduction or early termination of chemotherapy. Patients who received neoadjuvant paclitaxel (i.e. chemotherapy prior to surgery) were excluded from the study. Clinical data on patient demographics, tumor characteristics, paclitaxel dosing and toxicities was collected retrospectively from the medical records. Neuropathy data was recorded based on physician-documented notes and graded according to the National Cancer Institute Common Toxicity Criteria for Adverse Events (NCI-CTCAE) version 4.0. To minimize physician variation of grading of neuropathy in this retrospective review, we defined “severe neuropathy” to have occurred in patients who required a dose reduction, dose delay or early termination of paclitaxel due to paclitaxel-induced neuropathy.

### NDRG1 expression in normal nerve tissue and correlation with paclitaxel-induced neuropathy

Archival pathological specimens of resected breast cancer with corresponding nipple sections were retrieved. Nipple sections were chosen, due to the abundance of normal nerve tissue found within these sections. NDRG1 and S100 protein immunohistochemistry (IHC) staining was performed on consecutive sections of formalin-fixed, paraffin-embedded tissue blocks using a rabbit monoclonal antibody for NDRG1 (#5196, Cell Signalling Technology, USA) and S-100 protein (polyclonal, rabbit, Antibody ID: AB_10013383) on the Leica BOND-MAX autostainer in a histopathology core facility at the Cancer Science Institute, Singapore. NDRG1 was used at 1:50 dilution, while S100 was used at 1:500 dilution. NDRG1 IHC and S100 IHC has been previously validated [11, 12].

**Fig 1:**
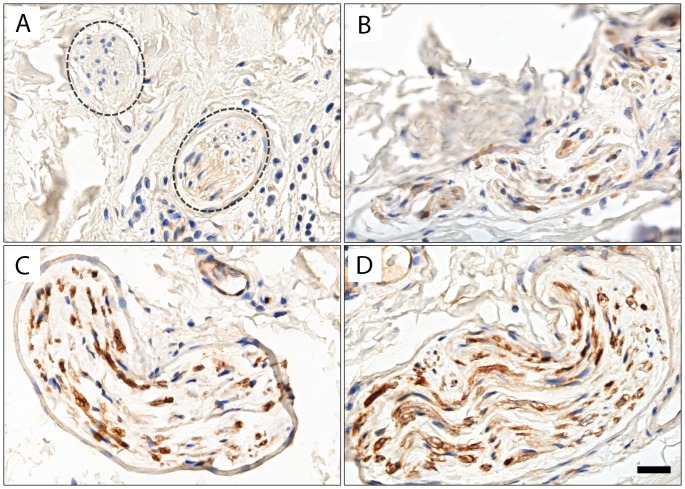
NDRG1 expression in normal nerve tissue. Photo micrographs (x 40 objective) depicting NDRG1 IHC, scale bar is 50μm. A: Score 0: Nerve highlighted by circles, with no expression of NDRG1. B: Score 1: Minimal expression of NDRG1 in less than 50% of the nerve bundle. C: Score 2: Strong expression of NDRG1 in less than 50% of the nerve bundle. D: Score 3: Strong expression of NDRG1 in more than 50% of the nerve bundle

NDRG1 cytoplasmic reactivity was assessed on one nerve bundle (highlighted by S100 staining) from each quadrant of each nipple tissue section according to the following method: Each nerve bundle was given a qualitative score of NDRG1 expression of 0 to 3 within nerve tissues. 0 was defined as no expression. 1+ as minimal expression in less than 50% of the nerve bundle, 2+ as strong expression, but not more than 50% of the nerve bundle or minimal expression in more than 50% of the nerve bundle, and 3+ as strong expression in more than 50% of the nerve bundle (Fig 1). The scores of all four nerve bundles in the four quadrants were then added to give a final NDRG1 expression score from 0 to 12. RS and BP scored each section separately and the final score was taken as the average of the two scores.

### Statistical Analysis

Predictive validity of NDRG1 was interrogated with receiver operating characteristic curve (ROC) analysis. NDRG1 scores between the two assessors was correlated using Spearman’s rank-order correlation test. Chi-square test and Fisher’s exact test were used to compare the occurrence of severe neuropathy with categorical variables such as ethnic group, presence of diabetes mellitus, and the cut-off values of NRDG1 from the ROC analysis. Differences in continuous variables such as age and NDRG1 IHC scores between patients who did or did not develop neuropathy was compared using the Mann-Whitney test. All tests were two-sided, with a 5% level of significance; all analyses were performed using SPSS v.20.0 (SPSS Inc., Chicago, Illinois, USA).

## Results

111 subjects with early stage breast cancer were included in the study. Patient characteristics are listed in Table 1. Median age was 55 years (range 29 to 79). 68% (*n* = 75) were Chinese, 19% Malay, 10% Indian and 4% of other ethnicity. 14% had diabetes mellitus. 41% had HER2 positive breast cancer and received adjuvant trastuzumab along with paclitaxel. A mean total dose of 904 mg/m^2^ (range 240 to 960 mg/m^2^) paclitaxel was administered, at a median of 12 weeks (range 3–18 weeks). 69% (*n* = 77) developed all-grade peripheral neuropathy, of which 15% (*n* = 17) developed severe neuropathy requiring dose reduction, dose delay or early termination of paclitaxel. 43% (*n* = 48) developed grade 1 neuropathy, 14% (*n* = 15) grade 2 and 11% (*n* = 12) grade 3 and none grade 4 by CTCAE version 4. Peripheral neuropathy occurred before cycle 6 in 48% of those who developed neuropathy. Expectedly, patients with diabetes mellitus suffered more severe neuropathy (44% vs 11%, *p*<0.01). However, there were no differences in neuropathy amongst various age groups or races.

**Table 1:** Patient Characteristics.

	N = 111	N (%)
**Age at diagnosis**	Median	55
	Range	29–79
**Race**	Chinese	75 (68%)
	Malay	21 (19%)
	Indian	11 (10%)
	Others	4 (3%)
**Diabetes**		16 (14%)
**ER +**		70 (63%)
**Her2 +**		45 (41%)
**Stage**	I	3(3%)
	II	67(60%)
	III	41(37%)
**Mean cumulative paclitaxel dose**		904 mg/m^2^ (240–960)
**Median treatment duration**		12 weeks(3–18)

**Fig 2:**
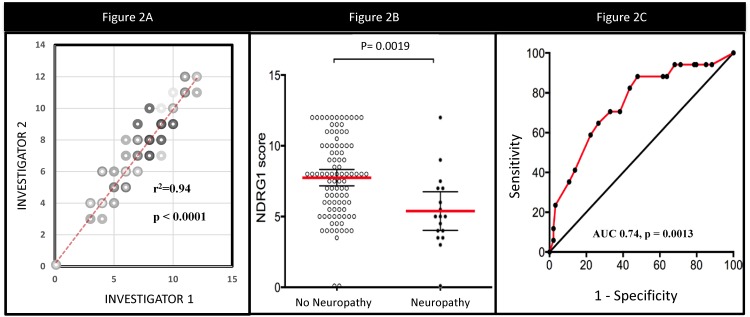
NDRG1 Analyses. 2A. Correlation of NDRG1 scores between investigator 1 and investigator. 2B. Mean NDRG1 expression scores in nerve tissue in subjects who did or did not develop paclitaxel-induced severe neuropathy. 2C. ROC analysis of predictive validity of NDRG1 for paclitaxel-induced severe neuropathy.

NDRG1 expression scores of the two assessors (RS, BP) were correlated, with an r^2^ of 0.94 (*p*<0.001) (Fig 2A). The average score between the two assessors was used as the final NDRG1 score for analysis. Mean NDRG1 score of patients without severe neuropathy was 7.7, while the mean NDRG1 score of patients with severe neuropathy was 5.4 (*p* = 0.0019) (Fig 2B). ROC analysis performed to determine the predictive validity of NDRG1 for severe neuropathy revealed an AUC of 0.74 (*p* = 0.0013), with a score of 7 yielding the highest sensitivity (77%) with the least loss of specificity (56%) (Fig 2C). 54 subjects had an NDRG1 score < = 7. Of these, 24% (*n* = 13) developed severe neuropathy, compared to only 7% (4/57) in those with a score >7 (*p* = 0.017). There was no correlation between nerve tissue NDRG1 score (< = 7 vs >7) with all grade neuropathy.

## Discussion

Paclitaxel-induced peripheral neuropathy is dose-dependent and its clinical manifestations are both sensory and motor. Modification of doses and early termination of chemotherapy remain the mainstay of managing paclitaxel-induced neuropathy. Existing treatment is only symptomatic, addressing pain, dysesthesia and paraesthesia [13]. The mechanism of paclitaxel-induced peripheral neuropathy is through the disruption of axonal microtubule dynamics, by binding to the β tubulin components of the microtubule assembly, leading to microtubule stabilization [14]. Other mechanisms of neuropathy include direct distal nerve terminal axonal toxicity inducing abnormalities in axonal mitochondria [15, 16]. The impact of paclitaxel-induced neuropathy is profound, leading to delays in curative treatment, and long term symptoms that are difficult to treat [17]. Studies have shown that healthcare costs for patients with neuropathy are higher than those without neuropathy and indirect costs may be significantly multiplied [18, 19]. To date, no biomarkers exist to predict for paclitaxel-induced neuropathy. We compared the level of NDRG1 expression in nerve tissue in patients who did or did not develop severe paclitaxel-induced neuropathy, and found patients with severe paclitaxel-induced neuropathy to have lower NDRG1 IHC scores in human nerve tissue. IHC testing on nerve tissues on previously resected surgical specimens can be readily performed, making this an appealing biomarker to develop that can have wide clinical applications.

NDRG1 is a part of the family of NDRG proteins (NDRG1 through NDRG4). NDRG1, a protein ubiquitously expressed in human tissues and tumors, has possible functions in growth arrest and cell differentiation and signaling protein shuttling between cytoplasm and nucleus [10, 20–23]. NDRG1 is mainly localized to the cytoplasm of myelinating Schwann cells and NDRG1 mutations in humans and mice are associated with demyelination [24, 25]. The high levels of NDRG1 expression in peripheral nerve and, specifically, in the Schwann cell, together with the characteristics of the CMT phenotype, point to a possible involvement of NDRG1 in Schwann-cell differentiation and the signaling necessary for axonal survival. Paclitaxel-induced peripheral neuropathy shares some of these characteristics, including sensory loss and secondary demyelination [5].

While there have been several SNP association studies published in the field of chemotherapy-induced neuropathy [26–28], none of these have found direct relevance or translation into clinical practice. Our findings suggest NDRG1 may be a potential novel biomarker for CIPN, with low NDRG1 expression in nerve tissue identifying patients at greater risk for paclitaxel-induced neuropathy for whom an alternative drug/regimen may be considered.

The main limitations of this study are its retrospective nature and its moderate sample size. Retrospective grading of neuropathy from clinician case notes is notoriously unreliable, but we have attempted to overcome this by defining a category of severe neuropathy using objective endpoints of dose reductions, dose delays or early termination of chemotherapy from neuropathy. Another limitation of a retrospective study is that included patients were not formally assessed with nerve conduction tests to determine if they already have pre-existing, non-chemotherapy related peripheral neuropathy at baseline. However, as early stage breast cancer patients tend to be younger with few co-morbidities, and clinicians are likely to consider a non-paclitaxel containing chemotherapy regimen in those with clinically significant pre-existing neuropathy, we believe it to be unlikely that the patients included in this study had pre-existing neuropathy. Validation of our findings would be required and we are now performing a larger prospective study to explore the mechanisms of NDRG1 regulation to support these promising findings.

## Supporting Information

S1 File NDRG1 study dataset. (PDF)
