# Supplementary material for: Low Levels of NDRG1 in Nerve Tissue Are Predictive of Severe Paclitaxel-Induced Neuropathy
Source: PLoS One. 2016 Oct 7;11(10):e0164319. doi: 10.1371/journal.pone.0164319 (PMC5055363; doi:10.1371/journal.pone.0164319)
Supplement: S1 File — (PDF) [file pone.0164319.s001.pdf]

| Pt No | NDRG1 investigator one | NDRG1 investigator two | NDRG1avg | Severe neuropathy | All grade Neuropathy | Age |
|---|---|---|---|---|---|---|
| 1 | 5.0 | 5.0 | 5.0 | 0 | 0 | 69 |
| 2 | 7.0 | 6.0 | 6.5 | 0 | 1 | 51 |
| 3 | 5.0 | 5.0 | 5.0 | 0 | 1 | 63 |
| 4 | 10.0 | 10.0 | 10.0 | 0 | 0 | 40 |
| 5 | 8.0 | 7.0 | 7.5 | 1 | 1 | 79 |
| 6 | 11.0 | 11.0 | 11.0 | 0 | 0 | 55 |
| 7 | 10.0 | 10.0 | 10.0 | 0 | 0 | 46 |
| 8 | 0.1 | 0.1 | 0.1 | 0 | 0 | 59 |
| 9 | 10.0 | 10.0 | 10.0 | 0 | 1 | 65 |
| 10 | 10.0 | 9.0 | 9.5 | 0 | 0 | 56 |
| 11 | 9.0 | 9.0 | 9.0 | 0 | 1 | 29 |
| 12 | 5.0 | 6.0 | 5.5 | 0 | 0 | 53 |
| 13 | 8.0 | 8.0 | 8.0 | 0 | 1 | 62 |
| 14 | 8.0 | 7.0 | 7.5 | 0 | 0 | 68 |
| 15 | 7.0 | 8.0 | 7.5 | 1 | 1 | 45 |
| 16 | 6.0 | 5.0 | 5.5 | 0 | 1 | 60 |
| 17 | 12.0 | 11.0 | 11.5 | 0 | 1 | 36 |
| 18 | 7.0 | 9.0 | 8.0 | 0 | 1 | 45 |
| 19 | 12.0 | 12.0 | 12.0 | 0 | 1 | 56 |
| 20 | 7.0 | 7.0 | 7.0 | 0 | 0 | 43 |
| 21 | 12.0 | 12.0 | 12.0 | 0 | 0 | 41 |
| 22 | 12.0 | 12.0 | 12.0 | 0 | 0 | 64 |
| 23 | 4.0 | 4.0 | 4.0 | 0 | 1 | 43 |
| 24 | 4.0 | 4.0 | 4.0 | 1 | 1 | 41 |
| 25 | 12.0 | 12.0 | 12.0 | 0 | 1 | 60 |
| 26 | 9.0 | 9.0 | 9.0 | 0 | 1 | 42 |
| 27 | 4.0 | 4.0 | 4.0 | 0 | 0 | 62 |
| 28 | 9.0 | 9.0 | 9.0 | 0 | 1 | 44 |
| 29 | 4.0 | 4.0 | 4.0 | 0 | 1 | 57 |
| 30 | 7.0 | 6.0 | 6.5 | 0 | 1 | 62 |
| 31 | 7.0 | 6.0 | 6.5 | 0 | 0 | 61 |
| 32 | 9.0 | 9.0 | 9.0 | 0 | 1 | 58 |
| 33 | 10.0 | 11.0 | 10.5 | 0 | 0 | 61 |
| 34 | 11.0 | 11.0 | 11.0 | 0 | 1 | 45 |
| 35 | 6.0 | 6.0 | 6.0 | 0 | 1 | 46 |
| 36 | 11.0 | 11.0 | 11.0 | 0 | 1 | 56 |
| 37 | 6.0 | 6.0 | 6.0 | 0 | 0 | 56 |
| 38 | 7.0 | 7.0 | 7.0 | 0 | 0 | 60 |
| 39 | 4.0 | 6.0 | 5.0 | 0 | 0 | 42 |
| 40 | 4.0 | 4.0 | 4.0 | 0 | 1 | 61 |
| 41 | 0.1 | 0.1 | 0.1 | 0 | 1 | 57 |
| 42 | 6.0 | 7.0 | 6.5 | 0 | 1 | 43 |
| 43 | 12.0 | 12.0 | 12.0 | 0 | 1 | 58 |
| 44 | 5.0 | 5.0 | 5.0 | 0 | 1 | 50 |
| 45 | 12.0 | 12.0 | 12.0 | 0 | 0 | 48 |
| 46 | 6.0 | 5.0 | 5.5 | 0 | 1 | 54 |

| | | | | | | |
|---|---|---|---|---|---|---|
| 47 | 5.0 | 5.0 | 5.0 | 1 | 1 | 67 |
| 48 | 5.0 | 5.0 | 5.0 | 0 | 1 | 54 |
| 49 | 6.0 | 6.0 | 6.0 | 0 | 1 | 45 |
| 50 | 5.0 | 5.0 | 5.0 | 0 | 0 | 38 |
| 51 | 8.0 | 8.0 | 8.0 | 0 | 0 | 56 |
| 52 | 7.0 | 7.0 | 7.0 | 0 | 0 | 75 |
| 53 | 7.0 | 8.0 | 7.5 | 0 | 0 | 47 |
| 54 | 9.0 | 10.0 | 9.5 | 0 | 0 | 74 |
| 55 | 8.0 | 8.0 | 8.0 | 0 | 0 | 57 |
| 56 | 12.0 | 12.0 | 12.0 | 1 | 1 | 51 |
| 57 | 12.0 | 12.0 | 12.0 | 0 | 0 | 51 |
| 58 | 12.0 | 12.0 | 12.0 | 0 | 0 | 64 |
| 59 | 8.0 | 8.0 | 8.0 | 0 | 1 | 49 |
| 60 | 8.0 | 10.0 | 9.0 | 1 | 1 | 42 |
| 61 | 8.0 | 8.0 | 8.0 | 0 | 1 | 37 |
| 62 | 11.0 | 11.0 | 11.0 | 0 | 0 | 40 |
| 63 | 5.0 | 6.0 | 5.5 | 1 | 1 | 50 |
| 64 | 5.0 | 5.0 | 5.0 | 1 | 1 | 47 |
| 65 | 7.0 | 6.0 | 6.5 | 0 | 1 | 64 |
| 66 | 6.0 | 8.0 | 7.0 | 1 | 1 | 65 |
| 67 | 6.0 | 6.0 | 6.0 | 0 | 1 | 52 |
| 68 | 5.0 | 5.0 | 5.0 | 0 | 1 | 47 |
| 69 | 8.0 | 8.0 | 8.0 | 0 | 1 | 38 |
| 70 | 5.0 | 4.0 | 4.5 | 0 | 1 | 54 |
| 71 | 3.0 | 4.0 | 3.5 | 0 | 1 | 47 |
| 72 | 9.0 | 7.0 | 8.0 | 0 | 1 | 57 |
| 73 | 7.0 | 8.0 | 7.5 | 0 | 1 | 73 |
| 74 | 8.0 | 8.0 | 8.0 | 0 | 1 | 51 |
| 75 | 10.0 | 10.0 | 10.0 | 0 | 0 | 40 |
| 76 | 5.0 | 4.0 | 4.5 | 0 | 1 | 60 |
| 77 | 11.0 | 11.0 | 11.0 | 0 | 1 | 54 |
| 78 | 6.0 | 6.0 | 6.0 | 0 | 1 | 70 |
| 79 | 0.1 | 0.1 | 0.1 | 1 | 1 | 65 |
| 80 | 6.0 | 5.0 | 5.5 | 0 | 1 | 51 |
| 81 | 4.0 | 4.0 | 4.0 | 0 | 1 | 46 |
| 82 | 8.0 | 8.0 | 8.0 | 0 | 1 | 60 |
| 83 | 10.0 | 10.0 | 10.0 | 0 | 1 | 49 |
| 84 | 3.0 | 3.0 | 3.0 | 1 | 1 | 67 |
| 85 | 5.0 | 5.0 | 5.0 | 1 | 1 | 52 |
| 86 | 3.0 | 4.0 | 3.5 | 1 | 1 | 75 |
| 87 | 4.0 | 3.0 | 3.5 | 1 | 1 | 64 |
| 88 | 8.0 | 7.0 | 7.5 | 0 | 1 | 41 |
| 89 | 5.0 | 4.0 | 4.5 | 0 | 0 | 56 |
| 90 | 12.0 | 12.0 | 12.0 | 0 | 1 | 59 |
| 91 | 4.0 | 4.0 | 4.0 | 0 | 1 | 40 |
| 92 | 4.0 | 4.0 | 4.0 | 0 | 0 | 54 |
| 93 | 8.0 | 8.0 | 8.0 | 0 | 1 | 48 |

| | | | | | | |
|---|---|---|---|---|---|---|
| 94 | 10.0 | 10.0 | 10.0 | 0 | 1 | 63 |
| 95 | 10.0 | 9.0 | 9.5 | 0 | 1 | 60 |
| 96 | 12.0 | 12.0 | 12.0 | 0 | 1 | 65 |
| 97 | 9.0 | 8.0 | 8.5 | 0 | 1 | 44 |
| 98 | 11.0 | 12.0 | 11.5 | 0 | 1 | 72 |
| 99 | 6.0 | 6.0 | 6.0 | 0 | 0 | 55 |
| 100 | 7.0 | 7.0 | 7.0 | 0 | 0 | 48 |
| 101 | 8.0 | 8.0 | 8.0 | 0 | 1 | 49 |
| 102 | 9.0 | 8.0 | 8.5 | 0 | 1 | 59 |
| 103 | 6.0 | 6.0 | 6.0 | 1 | 1 | 62 |
| 104 | 4.0 | 6.0 | 5.0 | 0 | 1 | 40 |
| 105 | 11.0 | 12.0 | 11.5 | 0 | 0 | 53 |
| 106 | 7.0 | 7.0 | 7.0 | 0 | 1 | 70 |
| 107 | 10.0 | 10.0 | 10.0 | 0 | 1 | 60 |
| 108 | 5.0 | 4.0 | 4.5 | 1 | 1 | 51 |
| 109 | 8.0 | 8.0 | 8.0 | 0 | 1 | 68 |
| 110 | 4.0 | 4.0 | 4.0 | 1 | 1 | 64 |
| 111 | 12.0 | 12.0 | 12.0 | 0 | 0 | 57 |

| Ethinicity | Diabetes | ER Status | Her2status | Cumulative dose of Paclitaxe |
|---|---|---|---|---|
| 1 | 0 | 1 | 0 | 960 |
| 1 | 0 | 0 | 0 | 960 |
| 1 | 0 | 0 | 1 | 960 |
| 1 | 0 | 1 | 0 | 960 |
| 1 | 0 | 0 | 1 | 240 |
| 1 | 0 | 1 | 1 | 960 |
| 3 | 0 | 0 | 0 | 960 |
| 3 | 0 | 1 | 0 | 960 |
| 1 | 0 | 1 | 0 | 960 |
| 2 | 0 | 1 | 1 | 960 |
| 2 | 0 | 0 | 1 | 960 |
| 1 | 0 | 0 | 1 | 960 |
| 3 | 0 | 1 | 0 | 960 |
| 1 | 0 | 0 | 1 | 960 |
| 1 | 0 | 1 | 0 | 832 |
| 1 | 0 | 0 | 1 | 960 |
| 4 | 0 | 0 | 0 | 960 |
| 1 | 0 | 0 | 1 | 960 |
| 1 | 0 | 1 | 1 | 960 |
| 1 | 0 | 0 | 1 | 960 |
| 2 | 0 | 1 | 1 | 960 |
| 1 | 0 | 1 | 1 | 960 |
| 1 | 0 | 0 | 0 | 960 |
| 3 | 1 | 0 | 0 | 880 |
| 1 | 0 | 1 | 0 | 960 |
| 2 | 0 | 1 | 0 | 960 |
| 1 | 0 | 1 | 0 | 960 |
| 1 | 0 | 0 | 0 | 960 |
| 1 | 0 | 1 | 1 | 960 |
| 1 | 0 | 1 | 1 | 896 |
| 1 | 0 | 1 | 1 | 880 |
| 1 | 0 | 0 | 1 | 960 |
| 2 | 1 | 1 | 0 | 960 |
| 4 | 0 | 0 | 1 | 960 |
| 1 | 0 | 0 | 0 | 960 |
| 1 | 0 | 0 | 1 | 960 |
| 1 | 0 | 1 | 0 | 960 |
| 1 | 0 | 0 | 0 | 560 |
| 1 | 0 | 0 | 1 | 240 |
| 1 | 0 | 1 | 0 | 960 |
| 2 | 0 | 1 | 0 | 960 |
| 1 | 0 | 0 | 0 | 960 |
| 2 | 0 | 1 | 0 | 880 |
| 2 | 1 | 0 | 0 | 960 |
| 1 | 0 | 0 | 0 | 960 |
| 1 | 0 | 1 | 1 | 856 |

| | | | | |
|---|---|---|---|---|
| 3 | 1 | 1 | 1 | 720 |
| 1 | 0 | 1 | 0 | 960 |
| 1 | 1 | 0 | 0 | 960 |
| 1 | 0 | 1 | 1 | 960 |
| 1 | 0 | 1 | 0 | 960 |
| 1 | 1 | 0 | 1 | 960 |
| 3 | 0 | 1 | 1 | 960 |
| 1 | 0 | 1 | 1 | 960 |
| 3 | 0 | 0 | 0 | 960 |
| 1 | 0 | 0 | 1 | 720 |
| 1 | 0 | 1 | 1 | 736 |
| 1 | 0 | 0 | 1 | 960 |
| 1 | 0 | 1 | 0 | 960 |
| 1 | 0 | 1 | 0 | 960 |
| 1 | 0 | 0 | 1 | 880 |
| 1 | 0 | 1 | 1 | 960 |
| 2 | 0 | 1 | 1 | 816 |
| 1 | 0 | 0 | 0 | 960 |
| 1 | 1 | 1 | 1 | 960 |
| 2 | 0 | 1 | 0 | 672 |
| 2 | 0 | 0 | 1 | 960 |
| 2 | 0 | 0 | 0 | 960 |
| 1 | 0 | 1 | 0 | 960 |
| 2 | 0 | 1 | 0 | 240 |
| 1 | 0 | 0 | 0 | 960 |
| 1 | 0 | 1 | 1 | 960 |
| 3 | 1 | 0 | 0 | 960 |
| 2 | 0 | 1 | 0 | 960 |
| 2 | 0 | 0 | 0 | 960 |
| 1 | 0 | 1 | 0 | 960 |
| 1 | 0 | 1 | 0 | 960 |
| 1 | 1 | 1 | 0 | 960 |
| 2 | 1 | 0 | 1 | 720 |
| 1 | 0 | 1 | 0 | 960 |
| 1 | 0 | 1 | 0 | 960 |
| 2 | 1 | 1 | 0 | 960 |
| 1 | 0 | 1 | 0 | 960 |
| 3 | 1 | 1 | 1 | 960 |
| 1 | 0 | 1 | 0 | 800 |
| 4 | 1 | 1 | 1 | 370 |
| 1 | 0 | 1 | 1 | 912 |
| 2 | 0 | 1 | 0 | 960 |
| 1 | 0 | 1 | 0 | 960 |
| 1 | 0 | 1 | 1 | 960 |
| 4 | 0 | 1 | 0 | 960 |
| 1 | 0 | 0 | 1 | 960 |
| 1 | 0 | 1 | 0 | 960 |

| | | | | |
|---|---|---|---|---|
| 1 | 0 | 1 | 0 | 960 |
| 1 | 0 | 1 | 0 | 960 |
| 1 | 0 | 1 | 1 | 960 |
| 1 | 0 | 1 | 0 | 960 |
| 1 | 0 | 0 | 0 | 848 |
| 1 | 0 | 1 | 0 | 960 |
| 1 | 0 | 1 | 0 | 960 |
| 2 | 0 | 1 | 0 | 960 |
| 1 | 0 | 1 | 0 | 960 |
| 3 | 1 | 1 | 0 | 800 |
| 2 | 1 | 1 | 0 | 960 |
| 1 | 0 | 1 | 0 | 960 |
| 2 | 0 | 1 | 0 | 928 |
| 1 | 0 | 1 | 0 | 960 |
| 3 | 1 | 0 | 1 | 720 |
| 1 | 0 | 1 | 0 | 960 |
| 1 | 0 | 0 | 1 | 655 |
| 1 | 0 | 0 | 1 | 960 |

| Total time for completion of treatment (weeks) |
|---|
| 12 |
| 13 |
| 12 |
| 12 |
| 3 |
| 12 |
| 12 |
| 12 |
| 12 |
| 12 |
| 12 |
| 12 |
| 13 |
| 12 |
| 13 |
| 12 |
| 12 |
| 14 |
| 13 |
| 12 |
| 15 |
| 15 |
| 12 |
| 13 |
| 16 |
| 12 |
| 13 |
| 12 |
| 12 |
| 14 |
| 12 |
| 12 |
| 14 |
| 12 |
| 12 |
| 14 |
| 12 |
| 10 |
| 3 |
| 12 |
| 12 |
| 12 |
| 14 |
| 12 |
| 12 |
| 13 |

| |
|---|
| 11 |
| 13 |
| 12 |
| 13 |
| 12 |
| 12 |
| 18 |
| 12 |
| 12 |
| 9 |
| 17 |
| 13 |
| 12 |
| 14 |
| 14 |
| 13 |
| 10 |
| 12 |
| 14 |
| 11 |
| 12 |
| 14 |
| 12 |
| 3 |
| 12 |
| 13 |
| 12 |
| 12 |
| 13 |
| 13 |
| 12 |
| 12 |
| 9 |
| 12 |
| 12 |
| 12 |
| 12 |
| 12 |
| 10 |
| 6 |
| 13 |
| 12 |
| 13 |
| 13 |
| 13 |
| 12 |
| 12 |

| |
|---|
| 12 |
| 12 |
| 12 |
| 13 |
| 15 |
| 12 |
| 12 |
| 13 |
| 12 |
| 11 |
| 13 |
| 12 |
| 13 |
| 15 |
| 9 |
| 12 |
| 8 |
| 12 |
